# Collinearity-based Assembly Correction Tool GUI: Software for collinearity-based genome assembly correction

**DOI:** 10.1093/g3journal/jkae277

**Published:** 2024-11-22

**Authors:** Shengcheng Zhang, Hejun Du, Xingtan Zhang, Binzhong Wang

**Affiliations:** Hubei Key Laboratory of Three Gorges Project for Conservation of Fishes, Chinese Sturgeon Research Institute, China Three Gorges Corporation, Yichang 443100, China; National Key Laboratory for Tropical Crop Breeding, Shenzhen Branch, Guangdong Laboratory for Lingnan Modern Agriculture, Genome Analysis Laboratory of the Ministry of Agriculture, Agricultural Genomics Institute at Shenzhen, Chinese Academy of Agricultural Sciences, Shenzhen 518120, China; Hubei Key Laboratory of Three Gorges Project for Conservation of Fishes, Chinese Sturgeon Research Institute, China Three Gorges Corporation, Yichang 443100, China; National Key Laboratory for Tropical Crop Breeding, Shenzhen Branch, Guangdong Laboratory for Lingnan Modern Agriculture, Genome Analysis Laboratory of the Ministry of Agriculture, Agricultural Genomics Institute at Shenzhen, Chinese Academy of Agricultural Sciences, Shenzhen 518120, China; Hubei Key Laboratory of Three Gorges Project for Conservation of Fishes, Chinese Sturgeon Research Institute, China Three Gorges Corporation, Yichang 443100, China

**Keywords:** genome assembly, manually correction, algorithm, Python software

## Abstract

Genome assembly errors can have a profound effect on downstream analyses. Collinearity-based Assembly Correction Tool GUI is designed to rectify these errors by leveraging collinearity information between the assembled genome and a reference genome. Collinearity-based Assembly Correction Tool GUI provides a user-friendly interface for visualizing and manually correcting assembly errors. It supports various operations such as insertion, deletion, inversion, and swapping of contigs and chromosomes. The software automatically reclusters, relabels, and redraws the assembly after each modification, ensuring that users can easily track changes. Collinearity-based Assembly Correction Tool GUI is a robust tool designed to efficiently correct large-scale assembly errors in polyploid genomes, featuring advanced collinearity detection capabilities.

## Introduction

Genome sequencing plays a crucial role in linking genotypes with phenotypic traits, while genome assembly is an essential procedure in genomic research (Verma *et al.* 2017). With the rapid advancement of sequencing technologies and the significant reduction in costs, genome assemblies at the contig-level genome have become increasingly common across numerous species. These genomic data have greatly enhanced our understanding of gene expression and regulation. As research into diploid genomes has reached a level of maturity, the focus has increasingly shifted toward tackling the more complex challenges presented by polyploid genomes. Concurrently, advancements in chromatin conformation capture technologies, such as 3C, 4C, and Hi-C, have significantly heightened interest in the spatial interactions of genes. However, contig-level genome assemblies are no longer sufficient to meet these research needs. As a result, constructing chromosome-level genomes has become imperative, particularly for studying polyploid species. In response to these challenges, a range of software tools has been developed to facilitate haplotype phasing. Tools such as WhatsHap (Patterson *et al.* 2015), HapCUT2 (Edge *et al.* 2017), Purge_haplotigs (Roach *et al.* 2018), Redundans (Pryszcz and Gabaldón 2016), and HaploMerger2 (Huang *et al.* 2017) employ sequence alignment techniques for haplotype phasing, whereas software like FALCON-Phase (Kronenberg *et al*. 2018), ALLHiC (Zhang *et al.* 2019), and HapHiC (Zeng *et al.* 2024) achieve this through assembly-based approaches. For chromosome anchoring, RaGOO (Alonge *et al.* 2019) and RagTag (Alonge *et al.* 2022) utilize reference genomes for this purpose, whereas LACHESIS (Burton *et al.* 2013), 3D-DNA (Dudchenko *et al.* 2017), ALLHiC, and HapHiC use Hi-C data. Additionally, polyploid genomes present greater assembly challenges compared with diploid genomes due to their larger size and higher complexity. Among the tools mentioned, only ALLHiC and HapHiC have successfully achieved both haplotype phasing and chromosome-level assembly for polyploid genomes.

Genome assemblies often contain errors stemming from sequencing inaccuracies, repetitive sequences, structural variations, and the high similarity between homologous chromosomes. These issues are particularly pronounced in polyploid genomes due to their increased number of haplotypes, which elevates the likelihood of assembly errors. To address phasing errors, several advanced techniques have been developed. A prominent approach involves trio-based methods, which leverage parental sequencing data. Additionally, anther sequencing-assisted assembly techniques have demonstrated promise (Sun *et al.* 2022). However, trio-based methods are limited to diploid phasing, and anther sequencing-assisted phasing incurs high costs and is not universally applicable. Our research group has developed the Prune function within the ALLHiC software, which significantly enhances phasing accuracy by filtering out erroneous Hi-C signals that cause contig grouping errors. Utilizing this method, ALLHiC has successfully assembled the sugarcane genome *Saccharum spontaneum* L. (Zhang *et al.* 2018) and has been applied to the phased assembly of several other genomes, demonstrating its broad applicability and effectiveness.

In practice, when phasing complex polyploid genomes, ALLHiC can still encounter contig grouping errors. These errors often appear as certain homoeologous groups containing multiple duplicated syntenic blocks, while others may lack specific syntenic blocks. Furthermore, errors in the ordering and orientation stages can result in synteny breaks and incorrect structural variations. These issues frequently necessitate manual segmentation and adjustment to achieve accurate assembly. Despite these challenges, ALLHiC remains an essential tool in polyploid genome assembly.

To address the challenges associated with manual correction in genome assembly, we developed the Collinearity-based Assembly correction Tool GUI (CATG). CATG is specifically designed to rectify issues related to the orders, orientations, and placements of contig clusters. This tool uses collinearity information to facilitate the manual correction of assembly errors, automatically inferring and visualizing problematic regions in genome assemblies to enable more intuitive corrections. Unlike Juicebox, which, despite its popularity, requires significant computational resources and extended running times (Durand *et al.* 2016), CATG adopts a distinct approach. While Juicebox relies on Hi-C signal data, CATG is anchored in collinearity-based analysis, providing users with an alternative method for genome assembly correction. CATG offers 3 distinct advantages: (1) clear haplotypic distinction. Utilizing synteny, CATG can clearly differentiate between different haplotypes of the same chromosome. In contrast, Juicebox requires manual identification of chromosome boundaries and haplotype groups based on existing Hi-C signals. (2) Resource efficiency. CATG operates with smaller files compared with Juicebox, which necessitates loading larger Hi-C signal and assembly files, resulting in lower resource consumption. (3) Ease of adjustment for complex genomes. For genomes with challenging phasing, Hi-C signals can be overly complex and difficult to adjust. CATG's synteny-based approach simplifies adjustments, making it more manageable. These advantages make CATG a superior tool for handling the complexities of genome assembly and phasing, particularly in resource-limited environments and with challenging genome structures. Therefore, CATG provides a more efficient solution by leveraging collinearity to detect and correct assembly issues, optimizing the genome assembly process without repetitive reassembly.

## Methods

**Fig. 1.**
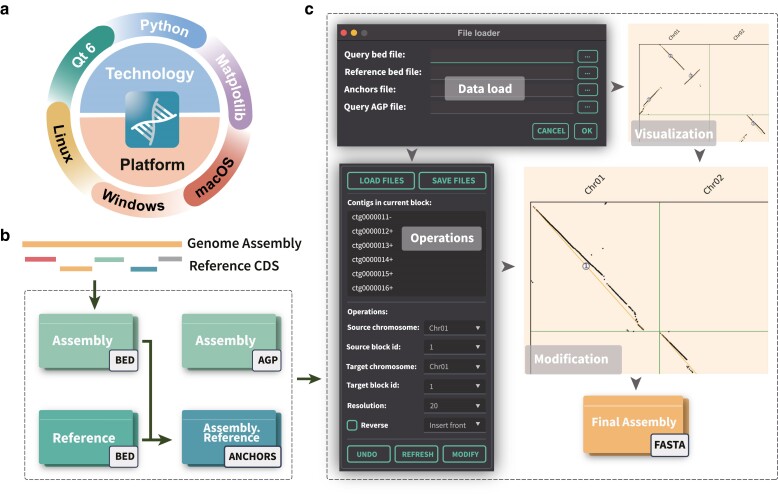
The overview of CATG and its workflow. a) CATG is an offline software platform developed using Qt6, Python, and Matplotlib and is compatible with Windows, Linux, and macOS operating systems. b) Input file preparation and required document generation workflow. First, the Assembly BED file is generated using genome assembly and reference coding sequences (CDS) data. Then, the anchors file is created by merging the Assembly BED file with the reference genome BED file. Finally, the assembled AGP file is obtained through the use of ALLHIC. c) CATG interface and execution workflow.

CATG was implemented as an offline cross-platform software (Fig. 1a) with its workflow illustrated in Fig. 1b and c. The software is implemented in Python 3, using the Qt framework for the graphical user interface and Matplotlib for visualizing collinearity and cluster plots.

### Data preparation and loading

The software requires 4 input data files. First, the Assembly AGP file is obtained from ALLHiC results. Then, coding sequences (CDS) are extracted from the reference genome and its annotations. These CDS sequences are aligned to the chromosome-level assembly of the target genome using GMAP, which provides gene location information and results in the Assembly BED file. To derive synteny information between the reference and target genomes, the JCVI software package is used with the Reference BED (from the reference genome) and the Assembly BED (from the target genome). This process generates the anchors file (Assembly.Reference.anchors).

### Visualization of synteny and clustering

Using the gene positions from the reference genome as the *y* coordinate and the positions from the target genome as the *x* coordinate, we plot the gene coordinates on a scatter plot. Setting a threshold of 1/*r* (where *r* is the resolution parameter) of the chromosome length, we calculate the distance between 2 genes. If the distance is less than the threshold, the genes are grouped into the same block. All blocks are sorted by the *x* coordinate, and any inflection point within a block—where the *y* coordinates of genes on either side of a gene are either all larger or all smaller than that of gene's *y* coordinate—indicates a split in the block. The resolution parameter configures the clustering of contig blocks, with higher values resulting in more blocks. We recommend a default value of 20 for genomes with less fragmentary collinearity. For genomes with significant fragmentary collinearity, increasing this value allows for more nuanced assembly adjustments. For each block, a line is drawn from the gene with the smallest *x* coordinate to the gene with the largest *x* coordinate, and the block is labeled with a block number (Algorithm 1).

**Algorithm 1.** CATG-Cluster gene list

**Input:** A list of gene “Genes”, each gene has 2 attributes: “xpos” and “ypos”
**Output:** Clusters of genes, “Clusters”
//First round of cluster
1: Sort(Genes);//Sort genes by xpos
2: Clusters = [[]];//Initialized as a double list
3: **For** *gene* in Genes **do**
4: **If** distance(*gene*, Clusters[−1][−1]) < threshold **then** //Calculate distance between the current gene and the last gene of the last cluster
5: Clusters[−1].append(*gene*); //If distance less than threshold, add the current gene to the last cluster
6: **Else**
7: Clusters.append([*gene*]); //Else create a new cluster, and add the current gene to it
8: **End if**
9. **End for**
//Second round of clustering
10: **For** *cluster* in Clusters:
11: **For** *i* **in** 1 to len(*cluster*)-1 **do** //For each cluster, evaluate the ypos of *gene_i_* relative to its neighboring genes
12: **If** *cluster*[*i*-1].*ypos* > *cluster*[*i*].*ypos* > *cluster*[*i* + 1].*ypos* or *cluster*[i-1].*ypos* < *cluster*[*i*].*ypos* < *cluster*[*i* + 1].*ypos* **then**
13: Divide(*cluster*, *i*); //If *gene_i_* has a higher or lower ypos than its neighbors, divide the cluster at *gene_i_*
14: **End if**
15: **End for**
16: **End for**
17: **Return** Clusters

### Position adjustment based on synteny

Using the AGP file and the gene positions on the target chromosome, we identify the contigs and their orientations within each block. When performing operations such as insertion, exchange, inversion, and deletion on blocks, we update the positions and orientations of the contigs on the target chromosome accordingly. This necessitates updating the gene positions on the target genome as well. After each adjustment, genes are reclustered, and a new synteny and clustering plot is generated. These operations are iteratively repeated until the synteny results meet the desired criteria.

### Acquisition of adjusted assembly results and saving adjustment plots

Upon completing the adjustments, the software generates a “tours” file, which records the order and orientation of contigs on each chromosome. Using this tours file, the ALLHiC_build module can produce the AGP and genome files. The final adjusted synteny clustering plot is saved as a PDF file.

## Results

**Fig. 2.**
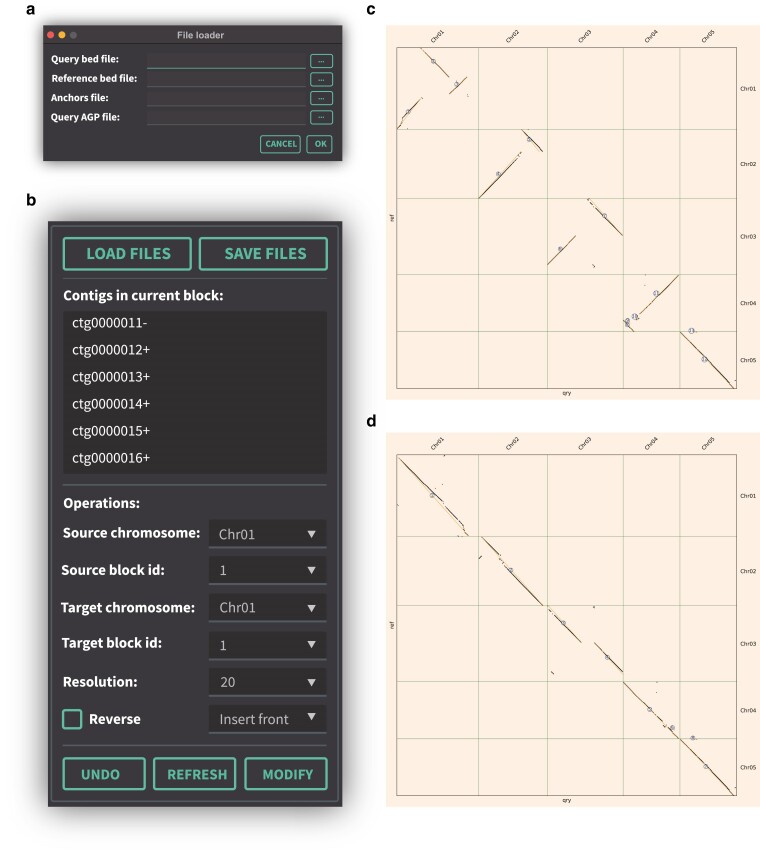
An example before and after error correction. a) File loader window. b) Contigs in current block panel (above) and operations panel (bottom). c) Collinearity plot before correction. d) Collinearity plot after correction.

We provided an example using a simple data, which required by CATG (https://github.com/sc-zhang/CATG/releases/download/v1.2.2/test_data.tar.gz), following operations should be taken. Figure 2c and d showed the ability of the manual correction.

### Data preparation and loading

Data are loaded into CATG by clicking the “LOAD FILES” button on the main interface, which will open the File Loader window (Fig. 2a). Files can be added either by dragging and dropping them into the designated text boxes or by using the “…” button to browse for files. The required input files include the following:

- The Query BED file and Reference BED file, which can be extracted from genome annotations using the JCVI software.
- The Anchors file, generated by the “jcvi.compara.catalog ortholog” function of JCVI software with “—cscore = 0.99” parameter.
- The Query AGP file, generated by the build function of ALLHiC software.

### Contig clustering and labeling

Following data loading, CATG automatically groups and clusters contigs aligned on chromosomes, visualizes these groupings, and assigns a corresponding ID to each cluster (Fig. 2c).

### Assembly correction operations

CATG supports a variety of manual correction operations, including:

- insertion: inserting a contig block before or after a target block or at the start or end of a chromosome,
- inversion: reversing the orientation of a chromosome or contig block,
- swapping: exchanging positions of chromosomes or contig blocks, and
- deletion: removing contig blocks from the assembly.

These operations are conducted via the “Operations” panel (Fig. 2b), where users select the source and target chromosomes and blocks, choose the desired operation, and click the “MODIFY” button. The software then automatically reclusters, relabels, and redraws the assembly after each modification (Fig. 2d).

As a reference-based tool for contig blocks assignment, several criteria must be considered. Contigs should be assigned to the linkage group that exhibits collinearity with them. In cases where a contig displays multiple collinearities with different linkage groups, often resulting from whole-genome duplication, it should be assigned to the linkage group exhibiting the strongest collinearity with best synteny score. For polyploids, contigs within the same chromosome group should maintain minimal overlap with each other.

### Viewing contigs within blocks

The “Contigs in current block” list dynamically displays the order and orientation of contigs within the selected block (Fig. 2b). This feature enables users to inspect and verify the assembly with enhanced resolution.

### Clustering at different resolutions

Users can adjust the resolution of contig clustering by specifying the resolution value in the “Operations” panel and clicking the “REFRESH” button (Fig. 2b). Increasing resolution values will produce a greater number of blocks, each containing fewer contigs.

### Undo functionality

To mitigate errors resulting from manual corrections, CATG includes a single-step undo function, allowing users to revert to the previous state by clicking the “UNDO” button (Fig. 2b).

### Saving results

The corrected assembly can be saved by clicking the “SAVE FILES” button and selecting a save location. CATG generates a corrected tour file and a collinearity plot, which can be converted to chromosome and AGP files using the build function of ALLHiC software.

This section may be divided by subheadings. It should provide a concise and precise description of the experimental results, their interpretation, and the experimental conclusions that can be drawn.

## Discussion

CATG is specifically designed to address the complexities of polyploid genomes. Its primary advantage over Hi-C-based tools, such as Juicebox, is its lower resource requirement, enabling rapid correction of large-scale assembly errors. CATG excels at efficiently correcting interchromosomal and interhaplotype contig positioning and orientation errors on a broad scale. However, CATG has its limitations. With advancements in sequencing technologies, contigs now possess sufficient length and accuracy to detect most real rearrangements. However, when genuine rearrangements occur between contigs, collinearity information alone is insufficient to confirm their authenticity. In such cases, additional data, such as Hi-C signals, are necessary to accurately distinguish real structural variations from assembly artifacts. Overall, CATG serves as a robust and efficient tool for large-scale genome assembly corrections, whereas Juicebox remains valuable for fine-tuning and local corrections. The strength of CATG lies in its advanced collinearity detection and correction algorithm, positioning it at the forefront of genome assembly technologies and providing researchers with a reliable and effective solution.

## Data Availability

All source code mentioned in this study can be found at https://github.com/sc-zhang/CATG (github) or https://zenodo.org/records/13621059 (DOI:10.5281/zenodo.13621059). The cross-platform packaged software could be found at https://github.com/sc-zhang/CATG/releases/tag/v1.2.2 (github) or https://zenodo.org/records/13621059 (DOI:10.5281/zenodo.13621059). The test data can be downloaded at https://github.com/sc-zhang/CATG/releases/download/v1.2.2/test_data.tar.gz (github) or https://zenodo.org/records/13621059/files/sc-zhang/CATG-v1.2.2.zip?download=1 (DOI:10.5281/zenodo.13621059) and can be found in test folder.
